# The role of soil in regulation of climate

**DOI:** 10.1098/rstb.2021.0084

**Published:** 2021-09-27

**Authors:** Rattan Lal, Curtis Monger, Luke Nave, Pete Smith

**Affiliations:** ^1^ CFAES Rattan Lal Center for Carbon Management and Sequestration, The Ohio State University, Columbus, OH 43210, USA; ^2^ Department of Plant and Environmental Sciences, New Mexico State University, Las Cruces, NM 88003, USA; ^3^ Biological Station and Department of Ecology and Evolutionary Biology, University of Michigan, Ann Arbor, MI 48104, USA; ^4^ Northern Institute of Applied Climate Science, United States Department of Agriculture Forest Service, Houghton, MI 49931, USA; ^5^ Institute of Biological and Environmental Sciences, University of Aberdeen, Aberdeen AB24 3UU, UK

**Keywords:** climate, soil carbon sequestration, soil inorganic carbon, forest soils, global warming, land-based solutions

## Abstract

The soil carbon (C) stock, comprising soil organic C (SOC) and soil inorganic C (SIC) and being the largest reservoir of the terrestrial biosphere, is a critical part of the global C cycle. Soil has been a source of greenhouse gases (GHGs) since the dawn of settled agriculture about 10 millenia ago. Soils of agricultural ecosystems are depleted of their SOC stocks and the magnitude of depletion is greater in those prone to accelerated erosion by water and wind and other degradation processes. Adoption of judicious land use and science-based management practices can lead to re-carbonization of depleted soils and make them a sink for atmospheric C. Soils in humid climates have potential to increase storage of SOC and those in arid and semiarid climates have potential to store both SOC and SIC. Payments to land managers for sequestration of C in soil, based on credible measurement of changes in soil C stocks at farm or landscape levels, are also important for promoting adoption of recommended land use and management practices. In conjunction with a rapid and aggressive reduction in GHG emissions across all sectors of the economy, sequestration of C in soil (and vegetation) can be an important negative emissions method for limiting global warming to 1.5 or 2°C

This article is part of the theme issue ‘The role of soils in delivering Nature's Contributions to People’.

## 1. Soils in the regulation of climate

The contribution of soils to the nature's contribution to people (NCP) ‘Regulation of Climate’ is controlled by the emission and sequestration of greenhouse gases (GHGs), biogenic volatile organic compounds and aerosols, and through impacts on biophysical feedbacks (e.g. albedo, evapotranspiration). Since soils contribute positively and negatively to each of these processes, evidence for each will briefly be summarized in §1 below, before examining in §2 how soils could be managed more effectively to maximize their contribution to this vital NCP, exploring what needs to be done to put this in to practice in §3, and providing some conclusions in §4.

### (a) Soils as a sink and source of atmospheric carbon dioxide

**Figure 1.**
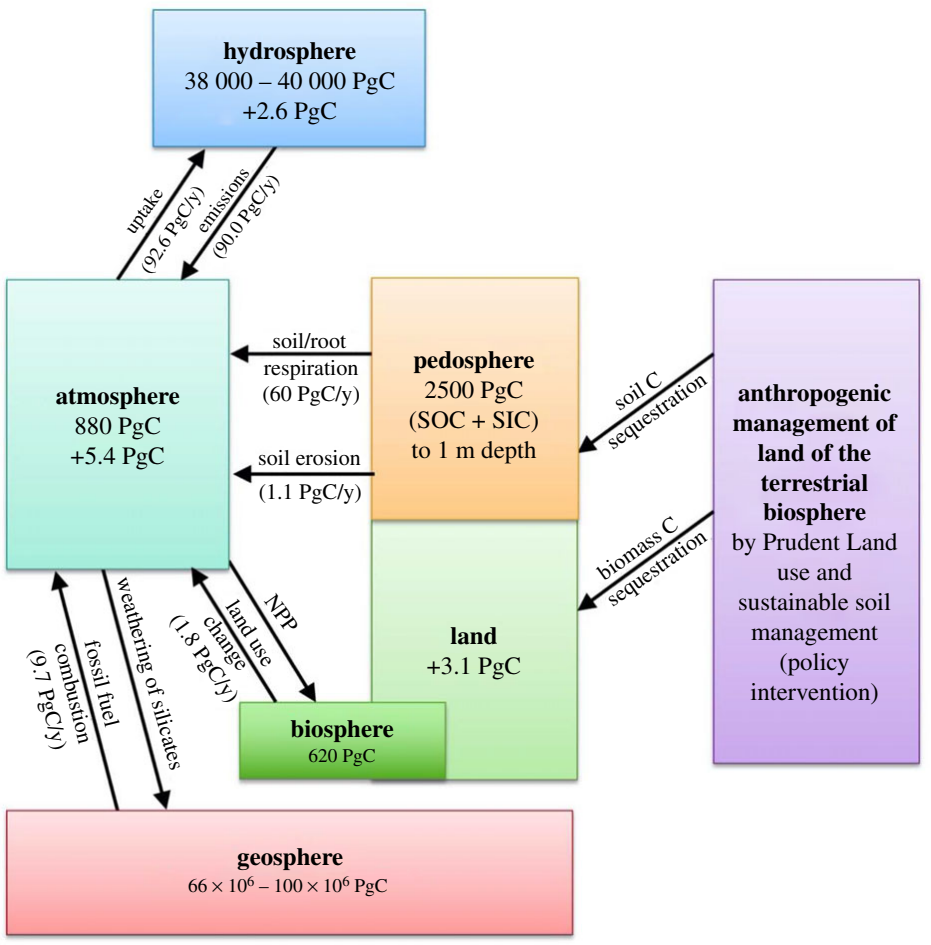
The role of soil and its management in moderating the global carbon cycle. The data on C stocks and fluxes are from [1–6].

Soils of the world constitute the largest reservoir of terrestrial carbon (C) stocks. They comprise both soil organic carbon (SOC) and soil inorganic carbon (SIC), and are an important component of the global C cycle (figure 1). Estimated to 1 m depth, terrestrial soil (2500 PgC; 1 PgC = petagram of carbon = 1 billion metric tons of carbon) and vegetation (620 PgC) hold three times more C than that in the atmosphere (880 PgC) [7]. However, estimates of soil C stocks are variable, depending on the methods used [8] (table 1).

**Table 1.** Differences in global and regional SOC stocks (PgC) to 1 m depth estimated by different methods. (Adapted and recalculated from Tifafi *et al*. [8].)

method	global	boreal	north temperate	tropical
soil grids	3421	1161	1376	865
HWSD-SOTWS	2439	390	890	1061
HWSD-SAXTON	2798	807	1237	696
average	2886	786	1168	874
difference between minimum and maximum	932	771	486	365
difference as % of the average	34	98	42	42

#### (i) Soil organic carbon

Current estimates of the global SOC stock range from 1500 to 2400 PgC [9]. However, SOC stocks are affected by temperature and precipitation, and there are concerns that projected climate change may destabilize SOC stocks, especially in regions of permafrost. With judicious management, however, SOC stocks are a critical component in keeping climate change under control (see §2). Mineralization of merely 10% of the SOC stock (estimated to be 1500 Pg to 1 m depth) is 15 times more than the 10 PgC emitted through fossil fuel combustion in 2019 [1]. On the other hand, land (soil and vegetation) currently absorbs about one-third of all anthropogenic emissions [1]. Assuming that the land-based C-sink capacity can be enhanced by adoption of judicious land use and prudent soil/crop management practices, harnessing the land-based sink offers a cost-effective option for adaptation to, and mitigation of, climate change. The attendant improvement in quality and functionality of soils of agroecosystems can accomplish the Agenda 2030 of the United Nations and advance several interrelated sustainable development goals [10].

Soils of agroecosystems have been a major source of CO_2_ ever since the dawn of settled agriculture. Ruddiman [11] estimated that the land use change from natural to managed ecosystems may have contributed as much as 320 PgC from the onset of agriculture (about 8000 BC) to circa 1750. The data in table 2 show estimated C emissions from land use change between 1750 and 2019. The data in table 2 indicate a decline in emissions from land use change as a percentage of the total anthropogenic emissions of 36–15%, because of the progressive increase in emissions from fossil fuel combustion, especially between 1960 and 2019. Regardless, data from land use change are incomplete because estimates of emissions are based on those owing to the loss and decomposition of biomass through deforestation, etc., but not considering the lateral transport owing to accelerated soil erosion, for example.

**Table 2.** Estimate of carbon emissions from land use change between 1750 and 2019. (Adapted and recalculated from Friedlingstein *et al*. [1]).

era	emissions (PgC)	% of the total	decade	emissions (PgC yr^−1^)	% of the total
1750–2019	255 ± 70	36.4	1960–1969	1.5 ± 0.7	33.3
1850–2014	200 ± 60	33.6	1970–1979	1.3 ± 0.7	22.0
1850–2019	210 ± 60	32.3	1980–1989	1.3 ± 0.7	19.4
1850–2020	85 ± 45	18.9	1990–1999	1.4 ± 0.7	18.4
1959–2019	210 ± 60	31.6	2000–2009	1.4 ± 0.7	15.4
			2010–2019	1.6 ± 0.7	14.6
			2019	1.8 ± 0.7	15.6

Forests and woodlands store a disproportionate share of the global SOC stock: they represent slightly less than 40% of global land area, but at approximately 400 Pg SOC, they store more than 45% of the SOC stock to 1 m [12–14]. Other estimates place forests and woodlands at 25–40% of global land area, with SOC stocks in the range of 400–800 PgC out of a global total of 1200–1600 Pg [15–17]. Global forest and woodland soils span a wide range in SOC densities, which was recently reviewed in the context of earth's global ecological zones (GEZs) [12–14]. Woodlands and shrublands in arid subtropical climates average less than 100 Mg SOC ha^−1^, while boreal and arctic forests and woodlands average nearly 600 Mg SOC ha^−1^. Arctic and boreal forests and woodlands cover approximately 30 million km^2^, which in combination with their large SOC density makes them the dominant component of the global forest SOC stock. Collectively, these soils represent more than 62% of global forest and woodland SOC on less than 37% of the global forest and woodland area. Although vastly distributed across regions with low human population densities, these ecosystems and their soils are not removed from vulnerability. Climate change and attendant increases in wildfire are significant sources of SOC vulnerability in the boreal zone [18–20]. Forest biomes in wet climates, such as the temperate oceanic, subtropical humid and tropical rainforests also have considerable SOC densities, in the range of 200–300 Mg SOC ha^−1^ [14]. Combined with their large extent (approx. 16 million km^2^), these wet biomes comprise 56 Pg SOC, or approximately 14% of global forest and woodland SOC stocks. Key climate and SOC management issues in these biomes also include increased wildfire, as well as land use pressures such as forest conversion to agricultural uses or plantations [21–25].

Forests and woodlands in biomes where they are not the dominant vegetation type are also important to the global SOC stock. Deserts, steppes and shrublands are the dominant vegetation types on over 58 million km^2^, or more than 72% of global land area. Nonetheless, forests and woodlands occupy approximately 13% of these lands. The limited areal extent and low SOC density of forests and woodlands in these dry biomes equate to only 12 Pg SOC (approx. 3% of the global forest and woodland total). However, wooded ecosystems are often disproportionately important providers of climate regulation and other ecosystem services in these dry biomes. In these biomes, especially in subtropical to tropical climates, subsistence uses long in equilibrium with forest and woodland dynamics have become increasingly challenged by climate change and demand for food, fibre and fuel resources [26–29].

#### (ii) Soil inorganic carbon

After SOC (*ca* 1526 PgC), SIC is the second largest pool of terrestrial C (*ca* 940 PgC), thus exceeding atmospheric C (*ca* 880 PgC) and land plants (549–615 PgC) [30–32]. Global stocks of SIC have been estimated at 780 PgC [33], 930 PgC [34], 695–748 PgC [35] and 940 PgC [36]. Because these estimates typically do not account for the SIC below 1.0 m depth, each estimate represents its own minimum amount and thus underestimates the actual amount. In addition to SIC as soil carbonate, the global amount of SIC as ${\mathrm{HCO}}_{3}^{-}$ in groundwater is at least 1404 PgC [37] with a global flux of 0.2–0.36 PgC yr^−1^ as ${\mathrm{HCO}}_{3}^{-}$ and a residence time as long as the residence time of groundwater itself, which may be hundreds to thousands of years [38–40].

**Figure 2.**
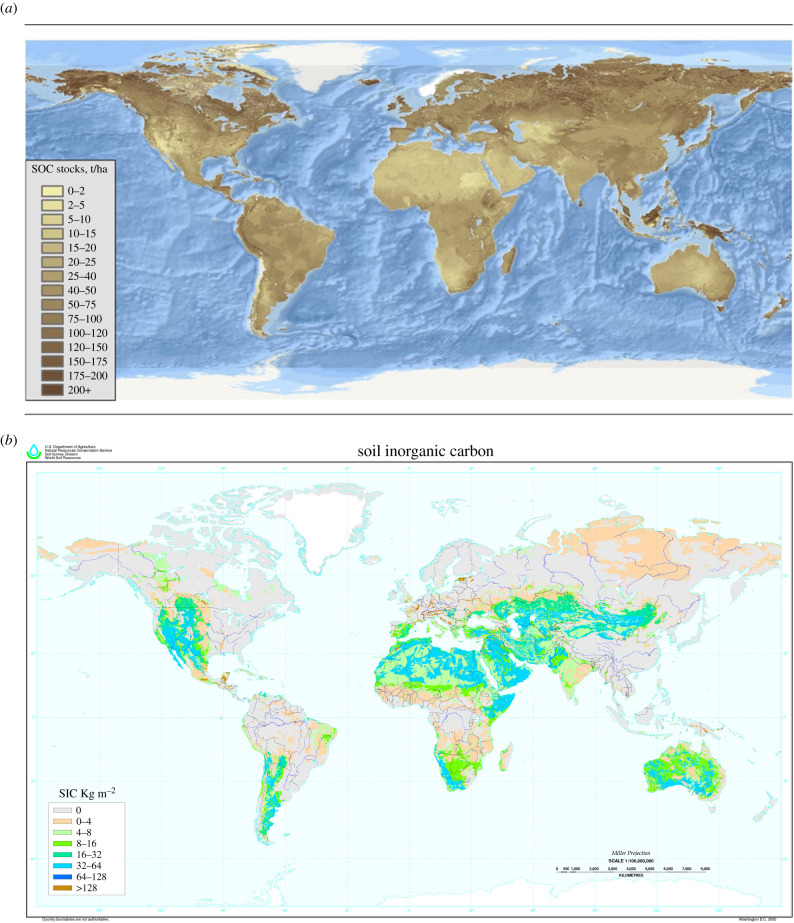
(*a*) Map of the global distribution of soil organic carbon (SOC) stocks. Produced by member countries under the guidance of the Intergovernmental Technical Panel on Soils and the Global Soil Partnership Secretariat, FAO, Rome. Tonnes per hectare (t/ha) = 0.1 kilograms per square metre (kg m^−2^). (*b*) Map of the global distribution of soil inorganic carbon (SIC) stocks. The SIC map is based on estimated carbon stocks to 1 m depth and a reclassification of the FAO-UNESCO Soil Map of the World [41] combined with a USDA-NRCS soil climate map [36]. Courtesy of USDA-NRCS, World Soil Resources, Washington D.C.

SOC and SIC often occur in the same soil. Unlike SOC, however, which exists in humid, semiarid and arid soils (figure 2*a*), SIC is mainly restricted to soils of arid and semiarid regions (figure 2*b*). Although SIC (as carbonates) can represent a substantial fraction in shrubland and grassland soils, forest soils are typically acidic and have little to no SIC [35,42].

**Figure 3.**
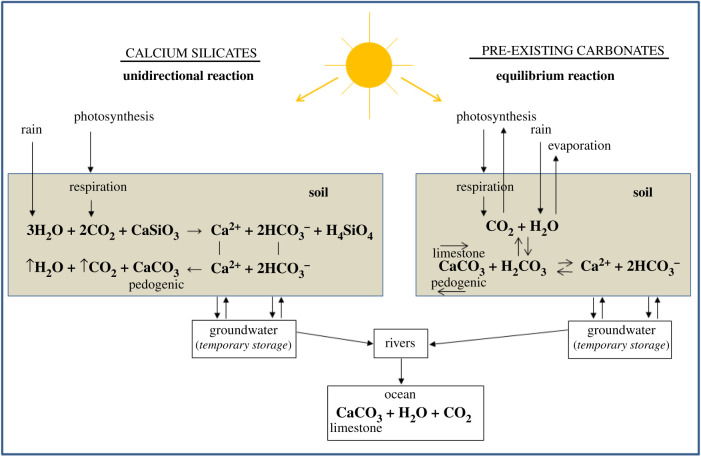
Soil inorganic carbon (SIC) pathways in soils and the hydrologic cycle contrasting the routes taken when calcium originates from silicates versus pre-existing carbonates.

SIC as used in this paper refers to the mineral phase, mainly calcite (CaCO_3_), of the carbonic acid system that also includes gaseous carbon dioxide (CO_2_), bicarbonate (${\mathrm{HCO}}_{3}^{-}$) and the carbonate ion (${\mathrm{CO}}_{3}^{2-}$). This system is the mechanism that enables CO_2_ to be pulled from the atmosphere and stored as CaCO_3_ in soil as bicarbonate in groundwater, and limestones in oceans (figure 3). Soil, therefore, is not only a C reservoir, it is also a bicarbonate generator (i.e. the medium in which chemically weathered silicate minerals produce bicarbonate). Thus, soil's role in regulating both short-term and long-term climate is paramount: short-term for producing pedogenic carbonate and bicarbonate in groundwater and long-term for producing limestone. The chemical weathering of Ca and Mg silicate minerals is the mechanism that controls the consumption of CO_2_ released by mantle degassing over geologic time, as shown by the Ebelman–Urey reaction [43,44]:

1.1 $${\mathrm{CO}}_{2}+{\mathrm{CaSiO}}_{3}\to {\mathrm{CaCO}}_{3}+{\mathrm{SiO}}_{2}.$$

Globally, carbon stocks of SOC and SIC are inversely related (figure 2*a*,*b*). In humid regions SOC is higher than SIC, while in arid regions SIC is higher. Arid regions contain roughly 78% of the global SIC, semiarid 14% and humid regions less than 1% [36]. The amount of SIC within arid regions is notably affected by three factors: extreme aridity, parent material and soil age [45]. In cases of extreme aridity (less than 50 mm of annual precipitation), for example, in the Atacama Desert of Chile, the Gobi Desert of Mongolia and the Mojave of the US, soils have lower CaCO_3_ amounts than deserts bordering steppes with greater rainfall (*ca* 250 mm), such as the Chihuahuan Desert [35,46]. Although calcareous dust and Ca^2+^ in rain can give rise to SIC regardless of parent material [47], in general parent materials high in Ca^2+^, such as limestone, give rise to soils with about twice the amount of soil CaCO_3_ than neighbouring parent materials low in Ca^2+^ [48].

Soil age within arid regions has important implications for carbon sequestration since progressively older soils contain progressively more SIC [49]. Although SOC can reach an equilibrium with its bioclimatic environment over decades to centuries, SIC can continue to accumulate C for thousands to tens-of-thousands of years as long as there is a supply of Ca^2+^ [50,51]. Thus, C can continue to be sequestered as SIC after SOC has reached its capacity.

Inventories of global stocks of SIC (e.g. figure 2*b*) do not differentiate between SIC precipitated in the soil profile (pedogenic) versus SIC existing as detrital particles of limestone (lithogenic) because routine laboratory methods, such as acid dissolution or dry combustion, cannot distinguish between the two types. In the field, however, pedogenic carbonate can be identified when carbonate crystals are organized into discrete bodies, such as filaments, nodules, pendants, subsoil horizons running parallel to the land surface and as petrocalcic horizons with laminar and plugged horizons [52]. At the microscopic scale, pedogenic carbonate can be identified when crystals are needle-shaped, have angular crystal faces, or occur as calcified root hairs, fungal hyphae or bacteria [53]. Lithogenic carbonate is relatively easy to identify if it occurs as stones and gravel but much harder to identify if it occurs as sand and silt unless microfossils are present [37].

### (b) Soils as a sink and source of non-CO_2_ greenhouse gases

Soils of agricultural and other managed ecosystems are also an important source of GHGs [21], including those of methane (CH_4_) and nitrous oxide (N_2_O), both of which are potent GHGs with 100-year global warming potentials of around 28 and 265, respectively [54]. In soils, N_2_O is generated mainly by the microbial transformation of nitrogen (N) under low oxygen conditions, and is dependent on the speciation of N, which varies mainly with pH [55]. This is often enhanced where available N exceeds immediate plant requirements, such as after fertilizer or residue application [56]. Methane (CH_4_) can be produced when organic materials decompose under low oxygen conditions in arable soils [57] with significant emissions from Histosols and flooded rice growing areas [58]. Cultivation of land for agriculture can significantly reduce the sink capacity of soils to oxidize CH_4_ [59]. Mineral soils under forests and other natural vegetation act as the strongest CH_4_ sink, followed by grasslands, with the sink strength weakest in cultivated soils and those receiving N fertilizer [59–61]; as such, as cropland has expanded, the CH_4_ sink strength of soils globally will have declined [59]. An objective of sustainable management of soil and agriculture is to reduce soil-based emissions of GHGs.

### (c) Other climate impacts of soils

Soils are not a significant source of biogenic volatile organic compounds or aerosols, but they are involved in biophysical climate feedbacks. In addition to their impacts on the global C cycle, and as a source or sink for CO_2_, CH_4_ and N_2_O, soils can exert other physical effects on climate through alteration of albedo and their influence on regional water cycles. The extent to which soils affect albedo is largely determined by how they influence the darkness of land surface, and whether they affect snow cover. Some soil amendments, such as biochar, darken the surface of soil and have been shown to reduce albedo [62–64], which it turn leads to some extent of climate warming. Other forms of management, for example leaving cereal straw on the soil surface, can increase albedo [64–67], thereby lowering their impact on climate warming. Since ploughed soils often lose more heat than untilled soils [68] and snow melts faster on tilled soils, ploughing may also exert indirect impact on albedo via its impact on snow cover, since snow cover leads to high albedo.

Soils are also important in regional water cycles [69], which may in turn impact evapotranspiration rates and sensible heat fluxes [70] and thereby affect to an extent local climate, though the impact of soils is difficult to quantify at larger scales. When soils are managed well to maximize SOC storage, they hold water better and are also more fertile [2,71]. This, in turn, may reduce the need for irrigation, and could reduce fertilizer needs. It will lead to reducing GHG emissions from pumping of irrigation water, and further reduce the embedded emissions in fertilizer production and direct emissions if less mineral fertilizer is applied to the soils (see §2).

## 2. Managing soils to better deliver regulation of climate

### (a) Increasing soil organic carbon sequestration

Soils can act as negative emission technologies (NETs) [64,72], also known as a carbon dioxide removal (CDR) option or a GHG removal option [73]. The most prominent NET is SOC sequestration. Sequestration of SOC is a three-step process: (i) photosynthesis of atmospheric CO_2_ into plant biomass-C, (ii) transfer of biomass-C into soil and its conversion into soil organic matter (SOM) and (iii) stabilization of SOM leading to increase in its mean residence time (MRT). Photosynthesis is often limited by deficiency of essential plant nutrients (especially N and P along with some micronutrients), and of plant available water (green water) supply in the root zone. The amount of biomass-C returned to soil of resource-poor small landholders is affected by the competing uses of crop residues for other purposes (e.g. feed for livestock, traditional biofuel) [74]. Conversion of biomass-C returned to the soil into SOM depends on the quality of biomass-C (e.g. C : N ratio, suberin content) and availability of nutrient elements in soil (i.e. N, P, S) [32].

The MRT of SOC depends on a wide range of factors [75], some of which are not well understood. Particle size distribution, and the amount and type (1 : 1 versus 2 : 1) of clay minerals are also critical in relation to the formation of stable microaggregates that can encapsulate SOM, decrease its accessibility to microbes [76] and affect the future of SOC. Another physical process of increasing MRT is the translocation of SOM from surface into the subsoil layers, and thus further away from the zone of intense agricultural and climatic perturbations. A chemical mechanism of enhancing MRT of SOM in soil is the formation of organo-mineral complexes and the role of polysacchrides [77] that decrease the rate of decomposition [32]. Decomposition of SOM by microbial processes is affected more by its accessibility than by its molecular structure [78], and that accessibility can be influenced by land use and management [32]. The objective of soil management for SOC sequestration is to create a positive soil/ecosystem C budget, whereby the input of C into soil (crop residues, cover crop biomass, manure, compost, biochar) is greater than the loss of C from soil (mineralization, erosion, leaching, fire).

Thus, soil and crop management practices important to creating a positive soil/ecosystem C budget include a system-based conservation agriculture or CA [79], and liberal input of organic manure and other amendments. A system-based CA encompasses a holistic approach and has key components including: (i) minimal soil disturbance or none, (ii) retention of crop residues on the soil surface as mulch, (iii) establishment of a cover crop during the off-season, (iv) adoption of complex rotations, (v) use of integrated systems of soil fertility management and (vi) integration of crops with trees and livestock. It is also important to realize that some manures can be a net source of GHGs and, thus, not as climate friendly as often assumed. Consequently, emission of all GHG must be considered in addition to soil C to identify practices that are truly net CO_2_ sinks.

Furthermore, losses of SOC must be minimized through adoption of conservation-effective measures, which reduce risks of accelerated erosion (i.e. water, wind, tillage). The technical potential of SOC sequestration has been assessed since the 1990s, and many of the available updates are cited in this article. In general, the potential of SOC sequestration is relatively more in cool and humid climates (0.5–1.0 MgC ha^−1^ yr^−1^) than that in agroecosystems of dryland regions (0.1–0.2 MgC ha^−1^ yr^−1^) ([2,23,32]; also see §3a for more updated references on this theme).

### (b) Increasing soil inorganic carbon sequestration

**Figure 4.**
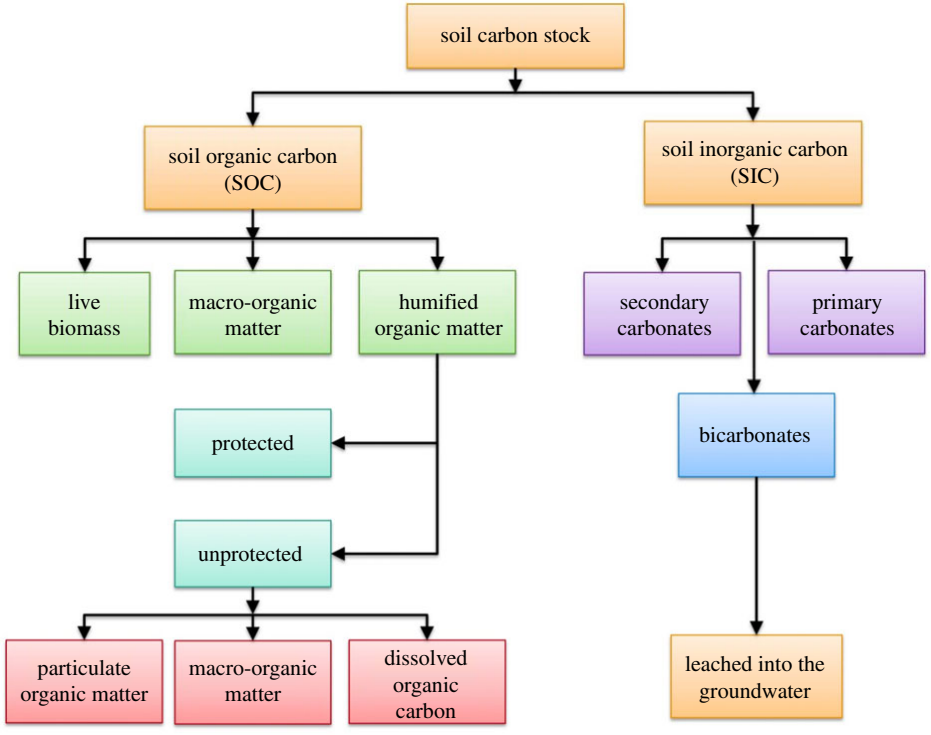
Components of the total soil carbon stock.

Identifying whether SIC is pedogenic or lithogenic is less important for understanding C sequestration by SIC than identifying the Ca^2+^ source. If Ca is directly from silicate minerals (i.e. ‘first generation’) and if SIC is pedogenic, then CO_2_ has been pulled from the atmosphere via the Ebelmen–Urey reaction. This unidirectional reaction not only represents long-term continental-scale weathering of silicates, it also represents short-term soil profile weathering and accumulation of pedogenic carbonates in ‘non-flushing’ soils of arid and semiarid climates. In its expanded form, the Ebelmen–Urey reaction can be used to track C sequestration in both soil and groundwater (figure 4). Two moles of CO_2_ react with one mole of Ca silicate (represented as CaSiO_3_), resulting in one mole of C sequestered as pedogenic CaCO_3_ and one mole of C released as CO_2_.

**Figure 5.**
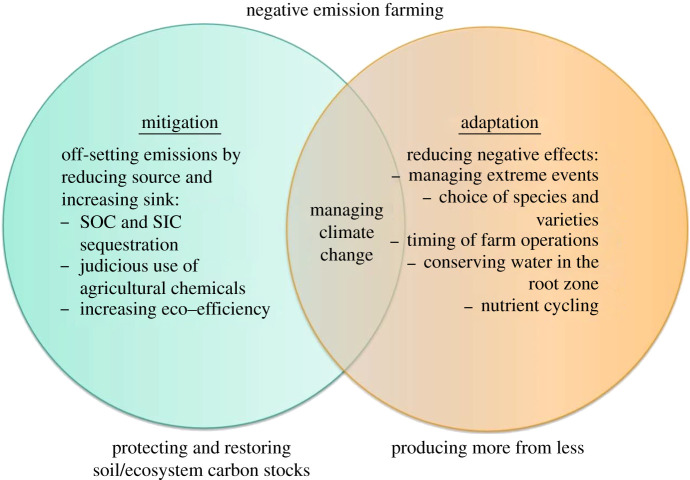
Strategies of mitigating and adapting to climate change and managing agroecosystems as a solution through transformation of food production systems.

If rainfall is sufficient, ${\mathrm{HCO}}_{3}^{-}$ is leached from soil into underlying aquifers where C is stored in groundwater. In this case, one mole of Ca^2+^ and two moles of ${\mathrm{HCO}}_{3}^{-}$ are stored. However, this is temporary storage that lasts until ${\mathrm{HCO}}_{3}^{-}$ combines with Ca^2+^ and precipitates as either (i) pedogenic CaCO_3_ if groundwater is pumped to the surface for irrigation or (ii) marine limestone if groundwater migrates into rivers and then oceans (figure 3).

Weathering of pre-existing carbonates, in contrast to weathering of silicates, is an equilibrium reaction in the form of a carbonate dissolution–reprecipitation (figure 3). In non-flushing soils of dry climates, limestone is dissolved by carbonic acid (H_2_CO_3_) and produces Ca^2+^ and $2\mathrm{HC}O_{3}^{-}$, which reprecipitate as pedogenic CaCO_3_ (figure 3). This reprecipitated CaCO_3_, however, does not sequester atmospheric C because the source of Ca^2+^ is from pre-existing CaCO_3_ and, thus, the CO_2_ that was consumed in the reaction to form carbonic acid is released upon the reprecipitation of CaCO_3_ [52].

In soils of humid climates, limestone is dissolved by carbonic acid and the Ca^2+^ and $2\mathrm{HC}O_{3}^{-}$ are transported to groundwater, which serves as a temporary pool for C sequestration. In karst terrain of China, for example, dissolution of limestone is estimated to sequester 12 Tg of C per year [80]. Eventually, Ca^2+^ and $2\mathrm{HC}O_{3}^{-}$ in groundwater are transported to streams and the oceans where they are biologically precipitated as limestone, upon which the impounded C from carbonate dissolution is released [44].

The process of SIC sequestration is primarily biological. Plant photosynthesis serves as a pump that brings CO_2_ into the soil, either directly via root respiration or indirectly via microbial decomposition of biological tissue. With no plants, the concentration of soil CO_2_ would equal the CO_2_ concentration of the atmosphere, thus slowing the reaction (equation (1.1)). In addition, plants exert controls on pH via carbonic acid as well as the formation of many other types of organic acids. Plants also exert strong controls on soil moisture and on Ca^2+^ availability, both of which effect the stoichiometry of the extended Ebelman–Urey reaction (figure 3).

A strong microbiological control of this process is also revealed by numerous studies showing an array of calcified bacteria, fungal hyphae and fine root hairs [81]. These field-specimen studies, combined with manipulative laboratory studies [82], provide evidence that under the right conditions, microorganisms precipitate calcite as biologically induced biomineralization, a form of biomineralization that results when organisms create extraneous environments conducive to CaCO_3_ formation [83]. Such is the case in arid and semiarid soils where microorganisms provide an aqueous micro-environment where Ca^2+^ and bicarbonate precipitate as CaCO_3_ in high pH environments [84].

### (c) Enhanced weathering

Another soil-related NET is enhanced weathering of silicate rocks (also known as accelerated weathering, with or without ‘rock’ or ‘mineral’ included). Enhanced weathering involves (i) the mining of rocks containing minerals that naturally lead to CO_2_ absorption from the atmosphere over geological timescales (as they become exposed to the atmosphere through geological weathering), (i) the grinding of these rocks to increase the surface area and (iii) the spreading of these crushed rocks on soils where they absorb atmospheric CO_2_ [85,86]. Construction waste, and waste materials (e.g. slag, overburden), can also be used as a source material for enhanced weathering.

In a systematic review of the costs and potentials of enhanced weathering, Fuss *et al*. [73] reported a wide range of potentials. The highest reported regional sequestration potential, 88.1 PgCO_2_ yr^−1^, is reported for the spreading of crushed rock over a very large surface area in the tropics [87]. The potential C removal on croplands only was estimated by Strefler *et al*. [88] to be 95 PgCO_2_ yr^−1^ for dunite and 4.9 PgCO_2_ yr^−1^ for basalt. Slightly lower potentials were estimated by Lenton [89], where the potential of C removal by enhanced weathering (including adding carbonate and olivine to both oceans and soils) was estimated to be 3.7 PgCO_2_ yr^–1^ by 2100, but with mean annual removal an order of magnitude less at 0.2 PgCO_2_-eq yr^–1^ [89]. Renforth & Campbell [90] [this issue] also cover aspects of enhanced weathering.

### (d) Other climate benefits from better soil management

When soils are managed well to maximize SOC storage, they have a higher water holding capacity [71], and are more fertile [2]. This, in turn, may reduce the need for irrigation and could reduce fertilizer needs, thereby reducing GHG emissions from pumping of irrigation water, and reducing the embedded emissions in fertilizer production and direct emissions if less mineral fertilizer is applied to the soils. Irrigation is energy intensive, with the energy for pumping often provided by fossil fuels, leading to a high emissions intensity. For example, El-Gafy & El-Bably [91] showed that pumping 1 m^3^ of water for an irrigated crop site in Egypt produces an average of 690 Mg CO_2_ per year. So, any reduction in requirement for irrigation by prudent soil management would deliver climate benefits.

As SOM decomposes, nutrients such as N are released, which could reduce the amount of fertilizer needed for food production. The default emission factor for direct N_2_O release from fertilization is 1 kg of N_2_O–N for every 100 kg N fertilizer applied, with additional indirect losses. Over a 100-year time horizon, one kg of N_2_O is around 265 times more potent than one kg of CO_2_ [92] in terms of climate warming. In addition to emissions from application in the field, emissions from fertilizer production add around 7–8 kg CO_2_-eq kg^−1^ fertilizer [93]. So, any reduction in the N fertilizer requirement of healthy soils will have great climate benefits. Judicious management of the soil not only contributes to mitigating climate change by reducing net emissions of GHGs (CO_2_, CH_4_, N_2_O), but it also contributes to adaptation to climate change by reducing its negative impacts (figure 5). Thus, judicious management of soils benefits adaptation to climate change by ‘producing more from less', enhancing eco-efficiency and reducing losses by erosion and other degradation processes (figure 5).

## 3. What is needed to put improved management of soils into practice?

### (a) Policy needs

Judicious management of the global C cycle has strong policy implications, especially with regards to managing soils of agricultural and forestry ecosystems (figure 4). Policy interventions are essential to encourage farmers/land managers to moderate the exchange of GHGs between soils and the atmosphere by adopting land use and soil/plant/animal management systems that create a positive soil/ecosystem C budget. The adoption of science-based and proven technologies by land managers can be promoted by political will and prudent governance through identification and implementation of policies at local, national, regional/continental and global levels. The importance of world soils has received the attention of policy makers since the launch of the ‘4 per 1000’ initiative at COP21 in 2015 in Paris [94]. Subsequent COPs (21 through 25) have supported similar initiatives at regional and global scales [95]. It is important, therefore, that soil scientists and agronomists seize the moment and support policymakers in translating science into action.

Payments to land managers for sequestration of atmospheric CO_2_ in soil (SOC and SIC) and in biomass (forest C-stock) would be a step in the right direction. Policies must be pro-farmer and pro-nature and specifically designed to enhance the land-based C sink (figure 4). The land-based C sink, estimated at 3.1 PgC in 2019 [1] (figure 4) or about 27% of the total anthropogenic emissions in 2019, can be enhanced through adoption of judicious land use and sustainable management of soils of managed ecosystems. The latter consist of a wide range of ecosystems including cropland, grazing/pasture/rangelands, forest/plantation land and urban lands. In addition, there are degraded soils and ecosystems that must be restored. Even in the U.S., the nation's Corn Belt has lost one-third of its topsoil [96] and the SOC stock's technical potential of C sequestration has been estimated at 1.27–3.66 PgC yr^−1^ (3.30 PgC yr^−1^) and that in the forest biomass at 2.0–4.6 PgC yr^−1^ (3.30 PgC yr^−1^) [3] (table 3). With use of biochar, the potential of SOC sequestration can be up to 3.20 PgC yr^−1^ [3]. Policy interventions are needed for protecting irrecoverable C in Earth's ecosystems [97], restoring degraded soils and desertified ecosystems by accomplishing land degradation neutrality [98] and managing C stocks in agriculture and forestry ecosystems [3]. In this regard, management and sequestration of SIC stocks in soils of arid and semiarid regions cannot be over-emphasized [37]. Policy interventions are also needed to spare land for nature, especially in developed countries [99], and through global adoption of integrated land use systems [100]. Biodiversity can be strengthened, and the terrestrial C stocks increased, if food is produced on a lesser area than the 5 billion hectares used at present [101]. Policy measures are also needed to set aside (retire) extremely and severely degraded lands. Globally, the area of such lands is estimated at approximately 390 Mha [102]. In addition, there are 700 Mha of peat lands (table 3) that must be protected.

**Table 3.** Potential of soil organic carbon (SOC) sequestration in soil and biomass of different agroecosystems (adapted from Lal *et al*. [3]). Note: Total technical potential C sequestration for the 80-year period 2020–2100 is 155 PgC in the biomass and 178 PgC in soils, or 333 PgC. This is equivalent to the drawdown of atmospheric CO_2_ of about 155 ppm [3]. Assuming that non-carbon fuel sources can take effect by 2050 or sooner, sequestration of C in the terrestrial biosphere can limit global warming to 2°C, if not 1.5°C.

land use	total area (10^6^ Mha)	sequestration rate (Mg C ha^−1^ yr^−1^)		total potential (Mg C ha^−1^ yr^−1^)
		biomass	soil	
cropland	1472	0.20–1.0	0.10–1.75	0.10–1.75
grazing land	3323	0.10–1.0	0.05–0.50	0.05–1.00
forest/woodland	980	0.20–2.0	0.15–1.00	0.15–2.00
urban lands	390	1.00–2.0	0.20–0.50	0.20–2.00
extremely/severely degraded lands	325	0.10–1.0	0.05–2.00	0.05–2.00
peatlands/wetlands	700	0.50–1.0	0.50–1.50	0.50–1.50
sub-total: degraded lands	1090			
total managed lands	7190			

### (b) Education needs

Education is needed to fully realize the beneficial roles that soils can play in the regulation of climate, and ecosystem services more broadly. The arenas of this education are fourfold: (i) soil science education and training for students and professionals; (ii) public outreach and education about the critical nature of soils to supporting life on Earth; (iii) education for all people in the ways that soils are connected with issues of equity and environmental justice; and (iv) education of policy makers to identify and implement appropriate policies to harness the land-based sinks.

The first of these educational spheres is the longstanding strength of the soil science discipline, and in many ways this is the easiest to sustain. Current understanding of the role of soils in climate regulation is the product of more than a century of academic and applied research, education and training in institutions of western scientific learning [103,104]. Experiential education related to soils and climate extends centuries further back, and lives on through the exchange of traditional soil and ecological knowledge [105,106]. But soil science education has not remained static. Compared to decades past, few today practice what could exclusively be called ‘soil science’; professionals in many disciplines use soil science tools and techniques in areas such as ecological sciences, geographical information systems and water resources management. Reaching these diverse disciplines has required ongoing re-evaluation and adjustment on the part of soils programmes and societies to ensure that soils education and training remain relevant and accessible [107,108]. These adjustments have included shifting away from traditional pedagogical approaches to alternative formats and practical or hands-on experiences. Targeted training events, such as those run by the US Forest Service International Programmes [109,110] or the Sustainable Wetlands for Mitigation and Adaptation Programme [111] provide efficient ways for students and professionals to learn how to apply soil science tools and techniques to soil quality, C and GHG accounting, and other efforts. Notably, the recent COVID-19 pandemic has enhanced some existing challenges and disparities in soils education, while also stimulating creative adaptation to online formats [112,113].

Education for the public, in order that all people are encouraged to examine and embrace our collective dependence on soils, is at least as important as the education of scientific and technical professionals. This need becomes all the more important as Earth's population continues to grow and urbanize, leading to ever larger numbers of people who lack direct connection to soils and their role in climate regulation, food production, water quality protection and the many other ecosystem services that they provide. However, the myriad ways in which humanity depends upon soils creates diverse opportunities to connect people to soils in individualized ways. The diversity of ways in which talented educators of our time are engaging in this work is impressive. From ‘Soil Kitchen’ events that provide real-time soil testing in urban communities [114], to joint US Forest Service-Tribal resource management workshops [115], to mainstream films [116] and magazine articles [117], soils educators and advocates are taking their message well beyond the realm of conferences, college classrooms and journal articles. Where soils education is taken into communities, rather than served from afar, it will continue to facilitate a wider societal appreciation for the ways that soils sustain us, and create opportunities for people to sustain them in turn.

To provide solutions to the climate crisis, soils education must address issues of equity and environmental justice. Indeed, soils, the climate crisis, and equity and environmental justice issues share a common theme: each is a nexus, a convergence of multiple interacting factors [118]. In the language of soil science, this nexus finds its name under the term ‘integrative,’ which recognizes that every unique soil body is the integration of many soil forming factors and processes. In the language of equity and environmental justice, this nexus is described by the ‘intersectionality’ of challenges faced by disadvantaged people and communities (or conversely, multiple intersecting forms of privilege). Women of colour in soil and Earth sciences experience both gender bias and racial discrimination; poverty-afflicted communities in urban areas experience the inequities not only of poverty and malnutrition, but also of metal-polluted soils and disproportionate climate change impacts. However, this intersection of challenges need not make them harder to resolve. On the contrary, addressing the barriers that inhibit any disadvantaged group in soil science can lower them for others, because the barriers are fundamentally often the same, such as structural exclusion, hostile behaviour and power imbalances [119,120]. Similarly, environmental justice movements can spur tangible actions such as urban composting and gardening that simultaneously address food security, soil pollution, C sequestration and climate change mitigation [121–124].

### (c) Research needs

Research is needed to develop better measurements, monitoring, standardization, upscaling from pedons to continents, identifying ecologically sensitive regions, understanding the biogeochemistry of terrestrial C, including black C, hydrophobicity and MRT in the context of land use and management [125]. For SIC, a supply of Ca^2+^ from silicates is essential for direct CO_2_ capture and storage as both pedogenic carbonate and enhanced weathering. Currently, ground basalt is the common source Ca^2+^. To remove one Pg of CO_2_ through enhanced weathering (reaction 1), approximately 3 Pg of basalt would have to be mined, crushed, and transported [88]. Research is needed to determine if more readily available forms of Ca^2+^, such as silicate-derived Ca^2+^ in gypsum or in calcium hydroxide, would be feasible.

Sequestration needs to be tailored to the environment where it will be implemented. Research is, therefore, needed to identify optimal areas using continental-scale ‘Land Resource Regions' or ‘Major Land Resource Areas [126]. Enhanced weathering, for example, will have a greater effect in low pH Ultisols than in neutral pH Mollisols. Given the large role of microorganisms in both SOC and SIC, such as the formation of pedogenic carbonates, additional research is needed that reveals the decomposition mechanisms and propensity of certain microbes for precipitating carbonate [127,128].

Research is also needed to determine the unintended consequences of geoengineering. This is especially relevant to manipulating the SIC system. An increase of 1% CaCO_3_ in global Mollisols, for example, from 8.25 to 9.25%, could sequester 14 Pg of C [37] over the time period that is required to increase the CaCO_3_.

## 4. Conclusion

The onset of agriculture circa 10 000 years ago [11] and that of the Industrial Revolution circa 1750 [1] have transformed the Earth and drastically disturbed the global C cycle. Notable among ramifications of the so-called ‘Anthropocene’ [129] that began with the onset of agriculture and accelerated with the Industrial Revolution are the following: soil degradation by erosion and other processes, depletion of terrestrial C-stock, an increase in atmospheric concentration of CO_2_ and other GHGs (CH_4_ and N_2_O) and attendant global warming, severe loss of biodiversity [130], as well as scarcity and eutrophication/contamination of natural waters [131]. Thus, there is a strong need to re-carbonize the terrestrial biosphere and restore C-stock in soil and forest biomass [3]. Sequestration of SOC and SIC in soil is a win–win option for mitigation and adaptation of global warming while restoring environmental quality and advancing sustainable development goals (SDGs) of the Agenda 2030 of the United Nations [132], while protecting C stocks of the natural ecosystems. It is critically important to restore those of the degraded and desertified lands, and to judiciously manage those of agricultural/forestry lands. Pro-farmer and pro-nature policies are needed to promote adoption of judicious land use and science-based management of soils/plants/animals to create a positive soil/ecosystem C budget [10]. In conjunction with replacing fossil fuels with non-C fuel sources, re-carbonization of soil and vegetation can limit global warming to 1.5 or 2°C.

## Data accessibility

This article has no additional data.
